# Inferior mesenteric artery diameter and number of patent lumbar arteries as factors associated with significant type 2 endoleak after infrarenal endovascular aneurysm repair

**DOI:** 10.1093/icvts/ivac016

**Published:** 2022-04-15

**Authors:** Stoyan Kondov, Aleksandar Dimov, Friedhelm Beyersdorf, Lars Maruschke, Jan-Steffen Pooth, Maximilian Kreibich, Klaus Kaier, Matthias Siepe, Martin Czerny, Bartosz Rylski

**Affiliations:** 1 Department of Cardiovascular Surgery, Faculty of Medicine, University Heart Centre, University Hospital Freiburg, Albert-Ludwigs-University of Freiburg, Freiburg, Germany; 2 Center of Diagnostic and interventional Radiology, St. Josefs Hospital, Freiburg, Germany; 3 Center for Medical Biometry and Informatics, University Medical Center, Freiburg, Germany

**Keywords:** EVAR, Type 2 endoleak, Coiling, Inferior mesenteric artery, Lumbar arteries

## Abstract

**OBJECTIVES:**

Our goal was to identify the inferior mesenteric artery diameter and number of patent lumbar arteries causing a significant type 2 endoleak to develop after infrarenal endovascular aneurysm repair.

**MATERIAL AND METHODS:**

Included were patients who underwent infrarenal endovascular aneurysm repair between April 2002 and January 2017. Patients with an aneurysm involving the iliac arteries were excluded. Significant type 2 endoleak was defined as a type 2 endoleak observed after infrarenal endovascular aneurysm repair and accompanied by abdominal aneurysm growth of at least 5 mm during that time.

**RESULTS:**

A total of 277 patients were included. Mean follow-up was 38.9 (standard deviation 121.6) months. Immediately after infrarenal endovascular aneurysm repair, type 2 endoleaks occurred in 55 patients (20%), resolving spontaneously in 2 patients 6 months after infrarenal endovascular aneurysm repair. Thirty (10.8%) patients revealed a significant type 2 endoleak with aneurysm sack enlargement > 5 mm during follow-up, for which inferior mesenteric artery or lumbar artery coiling was performed. Mean time for coiling after primary infrarenal endovascular aneurysm repair was 25.4 (standard deviation 19.10) months. Twenty-three patients (8.3%) showed a non-significant type 2 endoleak during follow-up (no aneurysm sack enlargement). We found that the inferior mesenteric artery diameter and number of patent lumbar arteries were factors associated with a significant type 2 endoleak (odds ratio 1.755, *P* = 0.001; odds ratio 1.717, *P* < 0.001, respectively). Prior to endovascular aneurysm repair, the inferior mesenteric artery was patent in 212 (76.5%) patients; its median diameter measured 3 (0.5–3.8) mm. The median number of patent lumbar arteries was 3 (2–4). According to our receiver operating characteristic curve analysis, an inferior mesenteric artery diameter ≥3 mm (sensitivity 93.3%, specificity 65%) and ≥3 patent lumbar arteries (sensitivity 87.5%, specificity 43.6%) proved to be optimal cut-off values related to developing a significant type 2 endoleak. We therefore propose a composite score for the development of a significant type 2 endoleak [(inferior mesenteric artery diameter + patent lumbar arteries)/2].

**CONCLUSIONS:**

Patients in whom the diameter of the inferior mesenteric artery is ≥ 3 mm and with ≥ 3 patent lumbar arteries carry a higher risk of developing significant type 2 endoleak after infrarenal endovascular aneurysm repair.

**Figure:**
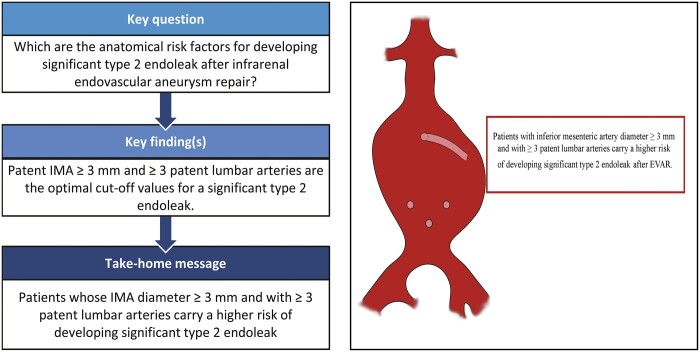

## INTRODUCTION

The goal of endovascular aortic repair (EVAR) is to exclude the aneurysm. However, it is not uncommon that blood flow persists within the aneurysm sac after EVAR—a phenomenon called endoleak. Although there is consensus about how to manage endoleak types I and III, what constitutes the best course of action for type 2 endoleak remains controversial [1].

A type 2 endoleak in patients presenting a stable aneurysm diameter is treated conservatively. But in case of a clinically significant type 2 endoleak accompanied by aneurysm sac growth, secondary interventions including inferior mesenteric artery (IMA) and the embolization or open surgery of the lumbar arteries are necessary to eliminate the risk of aneurysm rupture. Recent evidence indicates that the patency of the IMA and the number of lumbar arteries raise the risk for type 2 endoleak [2–4]. Embolizing the IMA or lumbar arteries before EVAR is a good option to prevent post-EVAR reinterventions. However, the incidence of type 2 endoleak lies between 7 and 23%; many of these are clinically insignificant and successfully treatable conservatively [5–8]. On the other hand, a type 2 endoleak is a condition leading in some patients to aneurysm sack growth and the risk of rupture [9]. Type 2 endoleak is an important issue in modern vascular surgery, and recently published series focused on preventive coiling in patients carrying a high risk for developing a type 2 endoleak [10, 11]. Identifying the factors associated with the development of a significant type 2 endoleak development would help us select patients for embolizing the IMA or lumbar arteries prior to EVAR, thus avoiding overtreatment [12, 13].

Our goal was to identify the number of patent lumbar arteries and the IMA diameter associated with an increased risk of developing a clinically significant type 2 endoleak after EVAR.

## METHODS

### Ethics statement

The ethical committee of the University Hospital Freiburg approved the study (561/19), and the informal agreement was waived due to the retrospective nature of the study.

#### Patient selection

Excluded are patients who underwent EVAR and concomitant endovascular treatment of the internal iliac arteries in order to create a more homogeneous group, emergency EVARs and patients with no computed tomographic angiography (CTA) follow-up. All patients were allocated to these 3 groups according to the type 2 endoleak observed after EVAR: no type 2 endoleak, non-significant type 2 endoleak and significant type 2 endoleak. A significant type 2 endoleak was defined as any endoleak observed after EVAR and accompanied by aneurysm sac growth of at least 5 mm compared to the diameter measured immediately after EVAR. Non-significant type 2 endoleak was defined as a type 2 endoleak accompanied by a stable aneurysm sac diameter (aortic diameter changes less than 3 mm within 6 months).

#### Endovascular protocol

EVAR was performed in patients with an anatomy suitable for infrarenal endovascular repair including an at least 15-mm proximal landing zone with an angulation less than 60° and 20 -mm distal landing zones. All EVARs were performed within the indication for use. All patients classified for EVAR were over 65 years old or had been deemed unfit for open repair. All procedures were conducted with the patient given general anaesthesia, with the surgical cut-down in the groin or percutaneous access. Heparin was administrated intravenously (100 IU/kg) before the endovascular procedure. All EVARs were conducted using the C-arm (Ziehm Imaging, Nuremberg, Germany). Protamine was administered at the end of the procedure, once all the wires and catheters had been removed from the vessels. Patients were extubated in the operating room. All patients received aspirin 100 mg orally daily beginning on postoperative day 1. If the patients received oral anticoagulation preoperatively and the EVAR was the only indication for aspirin, it was not given; the oral anticoagulation agent was continued postoperatively.

#### Imaging analysis

CTA (Siemens, Somatom, Erlangen Germany) was obtained at discharge, at 6 and 12 months and after EVAR, and yearly thereafter. Patients suffering from renal insufficiency underwent follow-up imaging via duplex sonography and were excluded from the study. Image analysis was performed by A.D. and S.K.

The diameter of the IMA at the orifice, the number of patent lumbar arteries, accessory renal arteries and the middle sacral artery were also evaluated on the patients’ preoperative CTA scans. Because we rarely observed lumbar arteries or a middle sacral artery with a diameter exceeding 2 mm, we determined the number of patent lumbar branches, not their diameter. Patent vessels are considered as those originating from the aneurysm sack that remain uncovered after EVAR. Because we often observed a stenosis at the origin of the IMA, we measured the minimal diameter of the artery because it is the smallest measured artery diameter that determines the flow through the vessel.

#### Coiling technique

Coiling was performed by deploying the vascular coils via the superior mesenteric artery or internal iliac artery.

#### Statistics

The statistical analyses were performed with SigmaPlot V 12.5 (Systat Software Inc., San Jose, CA, USA). Continuous variables were presented as the mean standard deviation (SD) in case of normal data distribution. If the normality test failed, data were presented as median with the 25^th^–75^th^ quartile. Continuous variables were tested for normality using the Kolmogorov-Smirnov test. Categorical variables were compared with the χ^2^ test. In case of small group sizes (*n* < 5), Fisher’s exact test was used. One-way analysis of variance (ANOVA) was used to identify significant differences when comparing the 3 continuous variables among the 3 groups. The Tukey test was applied for post hoc comparisons. In a case of not normal data distribution, the Kruskal-Wallis test was used with Dunn’s post hoc test in case of significance. T-test was used to compare continuous variables in 2 groups with normal distribution; otherwise the Mann-Whitney U test was applied. Receiver operation characteristics (ROC) curves were used to determine the cut-offs for IMA diameter and the number of the patent lumbar arteries for developing a clinically significant type 2 endoleak. Univariable logistic regression was performed using endoleak as a dependent variable to find the factors associated with clinically significant type 2 and, afterwards, multivariable logistic regression including all variables with *P* < 0.2 to determine the significance of the variables for developing clinically significant type 2 endoleak. The transparent reporting of a multivariable prediction model for individual prognosis or diagnosis (TRIPOD) recommendations were considered for creating the multivariable model [14].

### Composite score

The composite score was built as a composite variable from the IMA diameter and the number of the patent side branches (IMA diameter  + patent lumbar arteries including the middle sacral artery) and the value was then divided by 2.

## RESULTS

### Patient characteristics

Among the 360 patients who underwent infrarenal EVAR, 60 underwent concomitant endovascular treatment of the internal iliac arteries; 8 underwent emergency EVAR for a ruptured infrarenal aortic aneurysm; and 15 patients had no CTA follow-up and were thus excluded from further analysis. Included in our study were 277 patients whom we allocated into 3 groups: no type 2 endoleak (*n* = 224), non-significant type 2 endoleak (*n* = 23) and significant type 2 endoleak (*n* = 30). Overall, 4 patients (1.4%) were under combination of antiplatelet and oral anticoagulation therapy for other reasons, and 8 patients (2.8%) were under double antiplatelet therapy. Clinical characteristics are shown in Supplemental Table 1 showing no intergroup differences. Mean follow-up was 38.9 (SD 121.6) months. Overall, 2.9% (*n* = 8) patients died during follow-up; only 2 of them had endoleak type 2 (and they were in the non-significant type 2 endoleak group). The mean diameter of the infrarenal aneurysm before EVAR was 56.7 (SD 10.4) mm. The IMA was patent in 212 (76.5%) patients, and its median diameter measured 3 (0.5–3.8) mm. The most frequent IMA diameter we observed ranged from 2 to 4 mm (Supplemental Fig. 1). The median number of patent lumbar arteries including the middle sacral artery was 3 (2–4; Supplemental Fig. 1). At least 1 lumbar artery was patent in 92% of patients (Supplemental Fig. 1). An accessory renal artery was observed in 11 patients (3 with a type 2 endoleak and in 8 without) and the middle sacral artery was persistent in 53 patients. We saw a strong correlation between the IMA diameter and the patent lumbar arteries (*P* < 0.001 Person) (Supplemental Fig. 3). The various stent graft systems applied during EVAR were distributed similarly between groups (Supplemental Table 1).

### Incidence of endoleaks

The first CTA scan after EVAR taken before discharge revealed 3 (1.1%) type-Ia endoleaks and 1 (0.4%) endoleak type 1 b. Endovascular treatment before discharge succeeded in all patients.

We diagnosed a type 2 endoleak in 55 patients (20%); it resolved spontaneously in 2 of them 6 months after EVAR. We observed no late type 2 endoleak that had not been diagnosed on the initial CTA after EVAR. A non-significant type 2 endoleak was observed in 23 (8%) patients, which resolved spontaneously in 21 patients 12 months after EVAR. In the group presenting a non-significant type 2 endoleak, their aneurysm sack before EVAR revealed a mean diameter 59.5 (SD 9.3) mm and shrank later during follow-up to 55.4 (SD 10.1) mm. We noted a clinically significant type 2 endoleak in 30 (11%) patients: Their mean aneurysm diameter before EVAR was 56.4 (SD 8.6) and at intervention, 60.3 (SD 9.8) mm. Patients with clinically significant type 2 endoleak underwent interventional embolization. The mean time for coiling after primary EVAR was 25.4 (SD 19.1) months. The IMA was embolized in 13 patients, as were the lumbar arteries in 15; the IMA and lumbar artery were embolized in 2 patients during a single session. One patient underwent open conversion after an endovascular attempt to treat a type 2 endoleak failed. IMA embolization was complicated in 1 patient by an iatrogenic dissection of the superior mesenteric artery repaired by local patch reconstruction via a laparotomy. There were no detectable type 2 endoleaks in CTA after coiling in all but 1 patient: in him, we first had to embolize the IMA and then the patent lumbar arteries. Another patient had to undergo IMA embolization twice to obtain a complete IMA thrombosis.

### Factors associated with developing a significant type 2 endoleak

**Figure 1:**
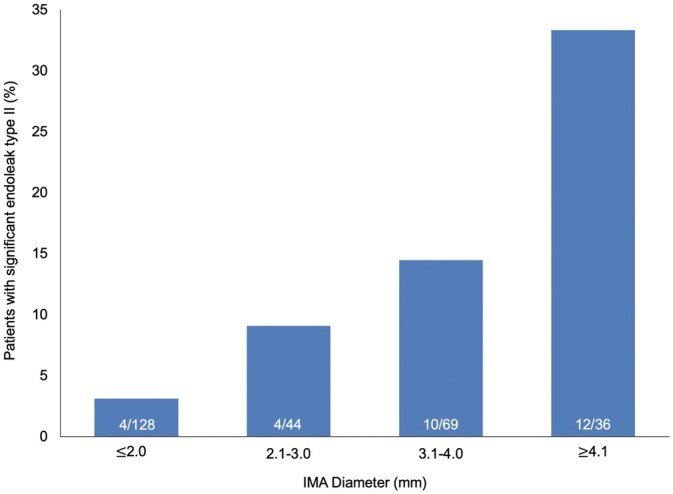
Inferior mesenteric artery diameter and number of patent lumbar arteries in patients undergoing EVAR. IMA: inferior mesenteric artery.

An increasing IMA diameter correlated with the incidence of significant type 2 endoleak. The highest incidence of significant type 2 endoleak was observed in patients with an IMA diameter measuring 3.1–4.0 and ≥4.1 mm (15% and 33%, respectively, Fig. 1).

**Figure 2:**
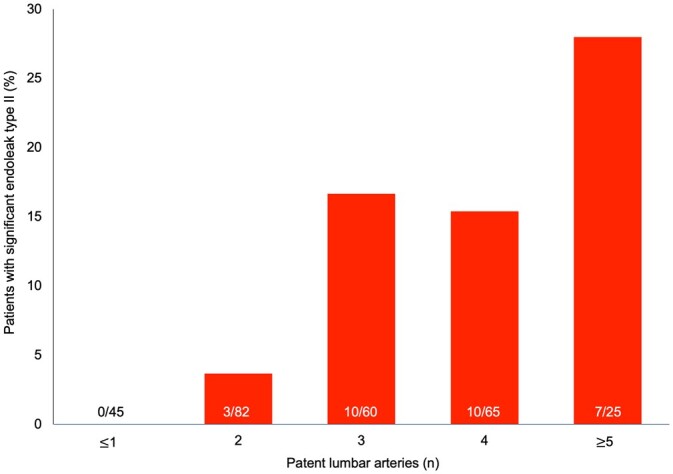
Number of patients presenting significant endoleak type II according to the inferior mesenteric artery diameter. Numbers at the bottom of the columns represent the number of patients with significant endoleak type II/number of all patients in the subgroup defined by inferior mesenteric artery diameter.

Higher numbers of patent lumbar arteries also correlated with the incidence of significant type 2 endoleak. We observed the highest rate of significant type 2 endoleak in patients with 3, 4 or ≥5 lumbar arteries (17%, 16% and 28%, respectively; Fig. 2).

Among 30 patients presenting a significant type 2 endoleak, all except 1 had a patent IMA. Nearly half (54.2% *n* = 150) had 3 or more patent lumbar arteries. Among all the patients included in our study, 36 (13%) had a patent IMA of at least 3 -mm diameter and 3 or more patent lumbar arteries. Only 4.9% (12/247) revealed an IMA ≥ 3 mm and ≥3 patent lumbar arteries that failed to develop a significant type 2 endoleak. The incidence of a large IMA (>3 mm) combined with >3 patent lumbar arteries was higher in the group with a significant type 2 endoleak than in the other patients: 80.0% (24/30) versus 4.9% (12/247), *P*<0.0001.

**Figure 3:**
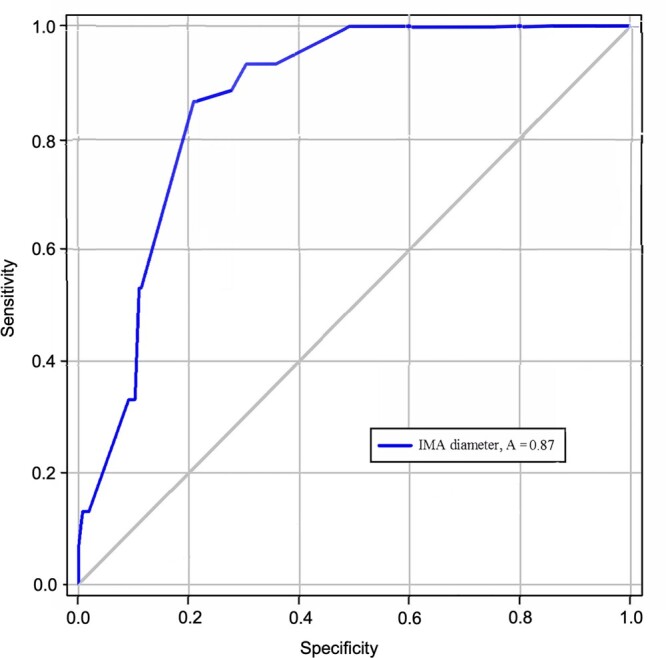
Number of patients with significant endoleak type II according to the number of patent lumbar arteries. Numbers at the bottom of the columns illustrate the number of patients with significant endoleak type II/the number of all patients in the subgroup defined by number of patent lumbar arteries. IMA: inferior mesenteric artery.

**Figure 4:**
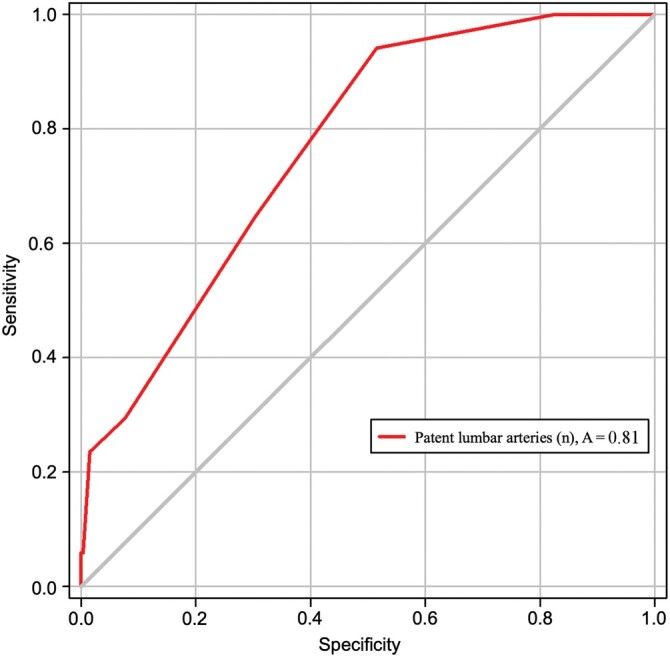
Receiver operating characteristics (ROC) curve analysis for the diameter of the inferior mesenteric artery relevant to endoleak type II, with an area under the ROC curve of 0.73 and nearest value to the upper left corner 3 mm with a sensitivity of 93.3% and a specificity of 65%. The closer the ROC curve is to the left upper corner, the higher is the test’s accuracy.

After performing univariable logistic regression all variables with P < 0.2 were included in the multivariable logistic regression model (Table 1). In our multivariable logistic regression analysis, the diameter of the IMA (odds ratio 1.755, 95% confidence interval 1.320–2.463, *P* = 0.001) and the number of open lumbar arteries (odds ratio 1.717, 95% confidence interval 1.264–2.387, *P* = 0.001) were associated with developing a significant type 2 endoleak (Table 2). A receiver operating characteristic **(**ROC) curve analysis was conducted to identify the cut-off value of the IMA diameter for developing a significant type 2 endoleak. The area under the ROC curve was 0.87, and a cut-off value of at least 3 mm IMA diameter revealed 93.3% sensitivity and 65% specificity, respectively (Fig. 3). Three patent lumbar arteries demonstrated a sensitivity and specificity of 87.5% and 43.6%, respectively (0.81 area under the curve) and were associated with developing significant type 2 endoleak (Fig. 4).

**Table 1:** Results of univariable logistic regression analysis of factors associated with clinically significant type 2 endoleak

Variables	95% Confidence interval	*P*-value
Age	−0.005-0.002	0.469
Sex	−0.103-0.167	0.642
Diabetes	−0.099-0.074	0.782
Hypertension	−0.133-0.150	0.901
Smoking	−0.136-0.021	0.151
AnaConDa ® stent graft system	−0.229-0.026	0.120
Endurant ® stent graft system	−0.275-0.058	0.201
Endurant IIs ® stent graft system	−0.247-0.033	0.195
Zenith ® stent graft system	−0.379-0.061	0.133
Aneurysm sack diameter	−0.002-0.005	0.509
IMA diameter	0.014-0.058	0.002
Patent lumbar arteries	0.016-0.069	0.002

IMA: inferior mesenteric artery.

**Table 2:** Results of multiple logistic regression analysis of factors associated with developing clinically significant type 2 endoleak detected at the 6-month computer tomography angiography follow-up

Variables	Odds ratio	95% Confidence interval	*P*-value
Smoking	0.672	0.270-1.587	0.203
AnaConDa ® stent graft system	0.680	0.221-2.353	0.602
Endurant IIs ® stent graft system	0.610	0.141-2.602	0.602
Zenith ® stent graft system	0.289	0.001-3.227	0.394
IMA diameter	1.755	1.320-2.463	0.001
Patent lumbar arteries	1.717	1.264-2.387	0.001

IMA: inferior mesenteric artery.

### Composite score

The composite variable was a mean score of 3.9 (SD 0.7) for the patients presenting a significant type 2 endoleak, 2.63 (SD 1.1) for those with a non-significant type 2 endoleak and 2.4 (SD 1.27) for the remaining patients (Supplemental Fig. 2). ROC curve analysis was performed for the composite score showing an area under the curve of 0.97 (Supplemental Fig. 4). The data for the specificity and sensitivity of the ROC curve analysis were presented in Supplemental Table 2.

## DISCUSSION

Our study findings can be summarized as follows:

- Prior to EVAR, the IMA was patent in most patients (77%), and the most frequently observed diameter measured between 2 and 4 mm. The majority of patients (92%) had at least 1 patent lumbar artery.
- A type 2 endoleak was observed in 20% of our patients after EVAR; we detected a clinically significant type 2 endoleak in 10.8%.
- An IMA diameter of ≥3 mm and ≥3 patent lumbar branches proved to be the optimal cut-offs for developing significant type 2 endoleak.

### Incidence of type 2 endoleak

The rate of type 2 endoleaks varies in the reported literature. Ward *et al.* published a series with 326 patients, with 30.4% (*n* = 99) showing a type 2 endoleak in postoperative CTA scans . Nevertheless, they focused on the general incidence and risk factors for type 2 endoleak but not on those leading to the development of type 2 endoleaks accompanied by aneurysm sack growth. Jones *et al.* reported an 18.9% type 2 endoleak in the first CTA 6 months after EVAR; however, the endoleak persisted in only 3.8% of the patients for > 6 months [5]. A large meta-analysis from Gelfand *et al.* reported an incidence of type 2 endoleak at discharge ranging from 6 to 17%, at 6 months after EVAR, 4.5 to 8% and at 1 year, 1 to 5% [15]. Twenty percent of the patients in our patient cohort revealed a type 2 endoleak—a percentage that prevails in the literature. In nearly half of them, coiling of the IMA or/and the lumbar arteries was performed, because the type 2 endoleak led to aneurysm sack growth.

### Management of type 2 endoleak after endovascular aneurysm repair

Managing type 2 endoleaks after EVAR is obviously difficult. The most common “watch and wait” approach includes EVAR and interventional embolization of the IMA and/or lumbar arteries, should significant aneurysm growth be observed at follow-up. Thanks to considerable progress in improving endovascular techniques, we have many new tools at our disposal for treating type 2 endoleak. Prophylactic or post-procedure embolization can be done with coils, vascular plugs, glue or ethylene-vinyl alcohol copolymer [16]. Additionally, there are other available methods such as endovascular IMA embolization via the superior mesenteric artery or the internal iliac artery as well as direct sac or transcaval puncture, all with durable results [17–20]. Nevertheless, coiling after EVAR could lead to frequent reinterventions, because they can lead to stent graft infection and kidney failure (through overly frequent contrast-agent application for repeated CTAs); they also demand more fluoroscopy time and may be associated with complications like the superior mesenteric artery dissection during post-EVAR IMA embolization we observed in this series. Range *et al.* showed that the combination of antiplatelet therapy with coumadin has the highest risk for the development of endoleak [21]. However, the authors did not analyze anatomical factors associated with the type 2 endoleak—only the antiplatelet and anticoagulation therapy. On the other hand, Wild *et al.* concluded that patients receiving warfarin and antiplatelet therapies do not exhibit increased incidence of postoperative endoleaks or aneurysm sac expansion after EVAR [22]. In 1 meta-analysis from 2017, antiplatelet therapy and oral anticoagulation were not associated with the onset of type 2 endoleak [23]. In the actual guidelines, there is no recommendation about the use of postoperative antiplatelets and anticoagulation regarding the type 2 endoleak after EVAR [1].

### Prophylactical coiling

On the other hand, coiling the IMA and lumbar arteries prior to EVAR has been suggested as a preventive measure. There is increasing evidence that anatomical risk factors predict the existence of a type 2 endoleak. Samura *et al.* showed that a patent IMA with ≥3 mm diameter (sensitivity 47.6% and specificity 85.4%) with at least 1 patent side branch ≥2 mm (sensitivity 85.7% and specificity 36.0%) are risk factors for type 2 endoleak [24]. Otsu *et al.* found that an IMA diameter of 2.6 mm (sensitivity 82.4% and specificity 65.6%) and lumbar branch diameters of 1.9 mm (sensitivity 80.0% and specificity 75%) increased the risk for developing type 2 endoleak [25]. Note that all those studies focused on determining risk factors leading to any type 2 endoleak development. Interestingly, the accessory renal artery was detected in 11 patients in our study, leading to the development of a type 2 endoleak in only 2 of them. Recently, it has been reported that prophylactic embolization based on anatomical criteria in patients at risk shows a significantly lower reintervention rate and significant aneurysm sac shrinkage after selective prophylactic coiling [10, 11]. There is a wide range of treatments of type 2 endoleak reported in these studies, which merely reflects the reality of type 2 endoleak management and its treatment. Although most of these studies applied similar methods, some diverge in various details, which could lead to different results.

### Identifying the significant type 2 endoleak

The novelty of our study is our intention to identify those patients presenting a risk for developing a clinically significant type 2 endoleak, because only those type 2 endoleaks require action beyond mere imaging follow-ups. Preventive IMA and coiling the lumbar arteries are unnecessary in patients at risk of developing a non-significant type 2 endoleak because those interventions can lead to overtreatment, since these patients present a diminishing aneurysm diameter despite having a type 2 endoleak and thus qualify for conservative therapy. We observed a type 2 endoleak in 20% of our patients immediately after EVAR. A spontaneous occlusion of a type 2 endoleak within the first 6 months after EVAR was observed infrequently. Among all our patients, the endoleak was clinically significant and triggered aneurysm-diameter growth in only 10.8%. We found that a higher number of patent lumbar arteries and a growing IMA diameter were associated with the risk for developing a significant type 2 endoleak. Our ROC curve analysis indicated an IMA diameter ≥3 mm and ≥3 patent lumbar branches as predictors for a significant type 2 endoleak. The specificity of the cut-off points is rather low and that could lead to false positive results. Therefore, we adopted the idea of creating a score consisting of the IMA diameter and the number of the patent lumbar arteries including the middle sacral artery to present the internal aneurysmal sack traffic in numerical fashion. Nevertheless, evaluation of such a composite score requires further evaluation in a prospective fashion. On the other hand, for patients with a non-significant type 2 endoleak, the “watch and wait” strategy makes sense, because their type 2 endoleaks resolved spontaneously in 21 of 23 patients. The higher incidence of a large IMA (>3 mm), together with the > 3 patent lumbar arteries we observed in the group with a clinically significant type 2 endoleak, supports the idea of prophylactic coiling in these patients. Nevertheless, some patients presenting these factors did not develop a significant type 2 endoleak. For them, prophylactic coiling prior to EVAR could signify type 2 endoleak overtreatment. Based on the literature and our findings, between 10 and 20% of patients with a type 2 endoleak could be suitable for prophylactic coiling [10, 11, 26,27]. Nevertheless, there is no published evidence regarding the prognosis of prophylactical coiling for a significant type 2 endoleak.

After analysing our data, we began prophylactic coiling in all those patients whose IMA diameter was ≥3 mm and who revealed ≥3 patent lumbar branches. Using the composite score, we suggest that combining the IMA diameter and patent side branches could help anticipate the risk for developing a clinically significant type 2 endoleak. Additionally, we saw a strong correlation between the number of the lumbar arteries and the IMA diameter. The both variables were the only 2 factors associated with developement of significant type 2 endoleak. The proposed score depicts the interference between the IMA diameter and the patent lumbar arteries. Obviously, before such a scoring system can be established, it will need to be prospectively validated through multicentric investigations.

Finally, identifying the patients at risk for developing a significant type 2 endoleak remains the key point, because our modern endovascular armamentarium provides a vast spectrum of modalities to treat them. A major, prospective multicentric trial could be the key to unifying the management and treatment of the type 2 endoleak and ensure their efficacy.

### Study limitations

This study has the following limitations: its monocentric, retrospective nature; no direct comparison with prospective IMA or lumbar artery coiling in patients at high risk for developing a type 2 endoleak; and the performance of the model has not been controlled for death using a competing risk analysis.

## CONCLUSIONS

The IMA and at least 1 lumbar artery are patent in the majority of patients undergoing EVAR. However, 20% may develop a type 2 endoleak after EVAR. Among all the type 2 endoleaks, every other one is clinically significant and leads to aneurysm-diameter growth. Patients presenting an IMA diameter of ≥3 mm and ≥3 patent lumbar branches proved to be carrying risk factors for developing a significant type 2 endoleak. The composite score could be useful for detecting patients at risk for developing a significant type 2 endoleak. Prophylactic embolization of the IMA and lumbar arteries in the appropriate patients is a potentially worthwhile strategy to minimize the later need for reinterventions.

## SUPPLEMENTARY MATERIAL

Supplementary material is available at ICVTS online.

### Data availability statement

All relevant data are within the manuscript and its supporting information files.

**Conflic of interest:** Martin Czerny and Bartosz Rylski are consul-tants to TerumoAortic and shareholders of AscenseMedical, Martin Czerny is consultant to Medtronic, Endospan and NEOS, received speaking honoraria from Cryolife-Jotec and Bentley and isshareholder of TEVAR Ltd.

## Supplementary Material

ivac016_Supplementary_Data

## Abbreviations

CI: Confidence interval
CTA: Computed tomographic angiography
EVAR: Endovascular aneurysm repair
IMA: Inferior mesenteric artery
ROC: Receiver operating characteristic
SD: Standard deviation
