# Supplementary figures and images for: Inferior mesenteric artery diameter and number of patent lumbar arteries as factors associated with significant type 2 endoleak after infrarenal endovascular aneurysm repair
Source: Interact Cardiovasc Thorac Surg. 2022 Apr 15;35(1):ivac016. doi: 10.1093/icvts/ivac016 (PMC9252125; doi:10.1093/icvts/ivac016)

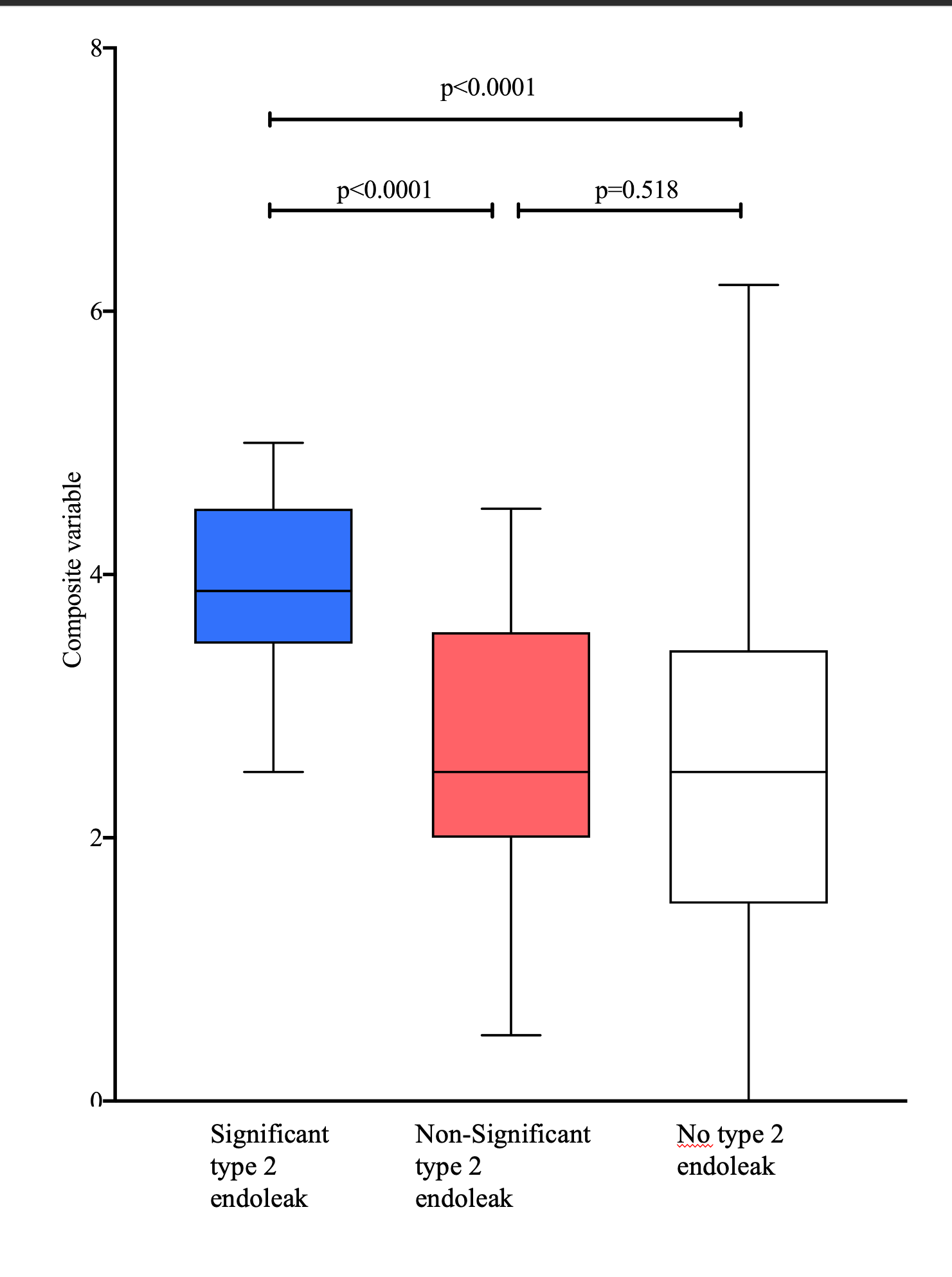

Supplement: ivac016_Supplementary_Data [file ivac016_supplementary_data.zip › Supplemental figure 2.png]

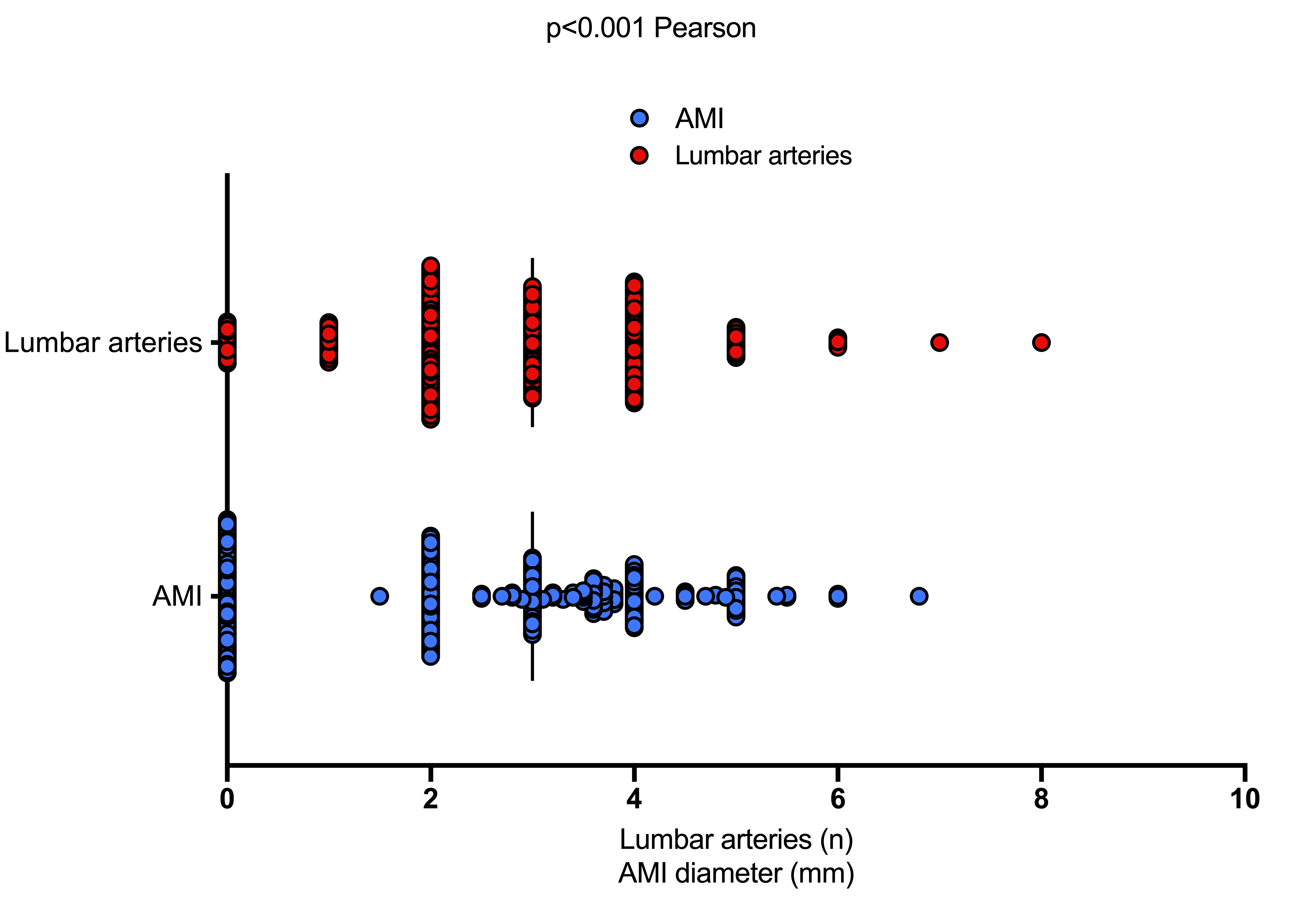

Supplement: ivac016_Supplementary_Data [file ivac016_supplementary_data.zip › Supplemental figure 3.png]

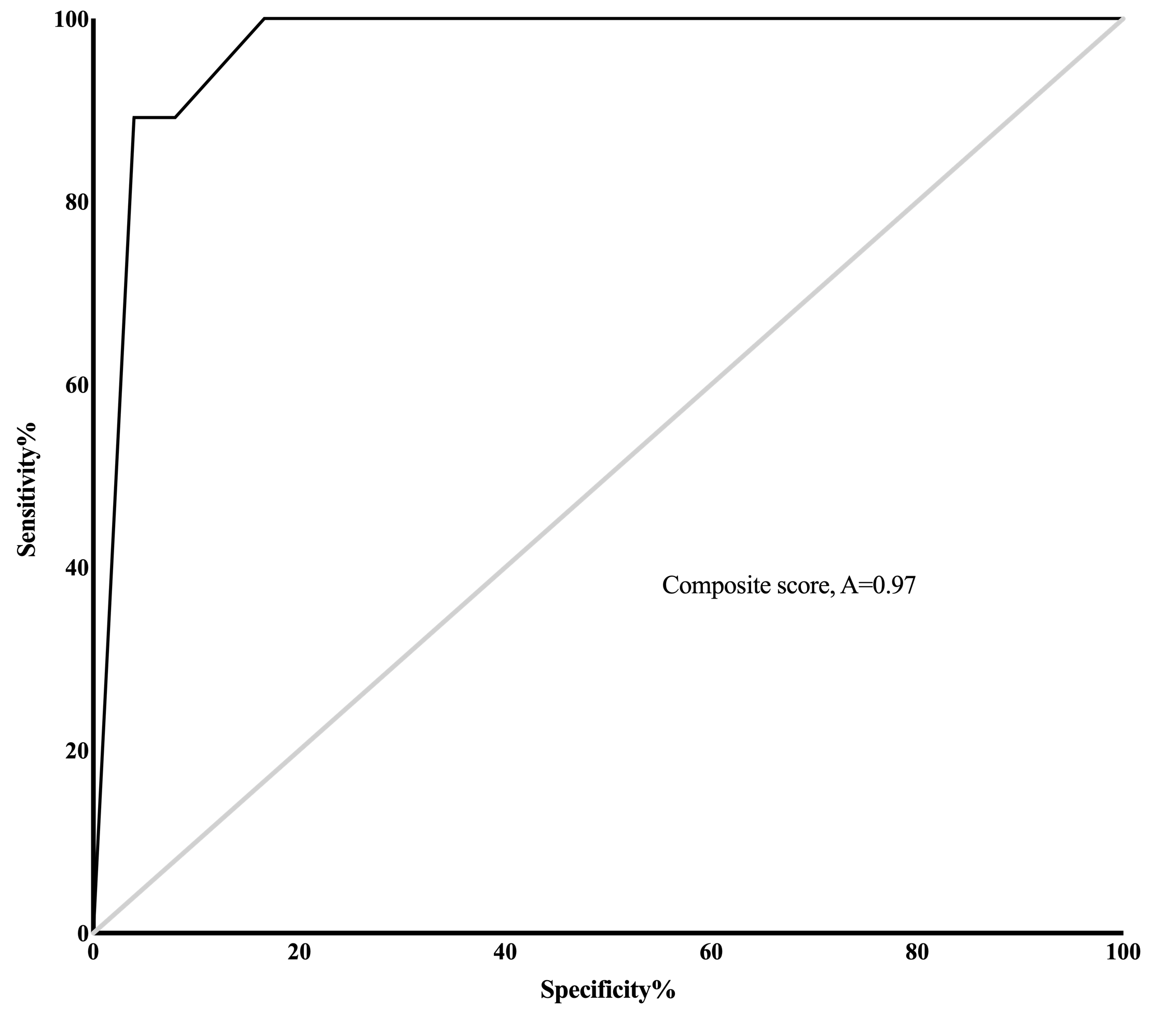

Supplement: ivac016_Supplementary_Data [file ivac016_supplementary_data.zip › Supplemental figure 4.png]

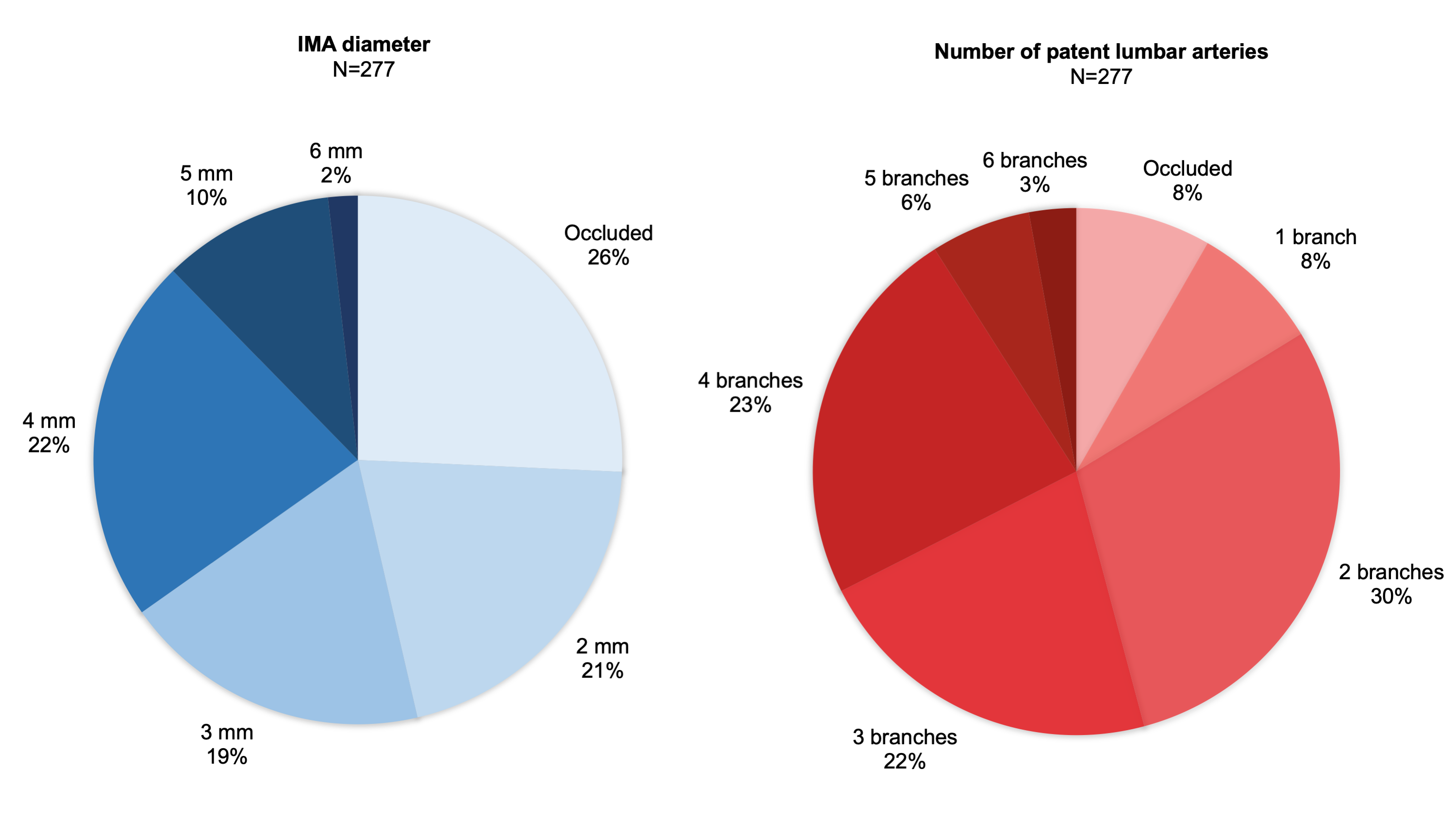

Supplement: ivac016_Supplementary_Data [file ivac016_supplementary_data.zip › Supplemental figure 1.png]
